# Imaging of Primary and Recurrent Tumours

**DOI:** 10.1080/135771402753667673

**Published:** 2002-05


### IMAGING OF PRIMARY AND RECURRENT TUMOURS

### ORAL PRESENTATIONS

### D41 The Role of Functional Nuclear Scanning in the Management of Cartilaginous Tumours

P. Choong[1,2], T. Kunisada[1], G. Powell[1,2], S. Schlict[1], R. Hicks[2], J. Slavin[1]
*[1]St. Vincent's Hospital, Melbourne, Australia, [2]Peter MacCallum Cancer Institute. Melbourne, Australia*

*Background:* Cartilaginous tumours may present diagnostic and prognostic dilemmas. Heterogeneity of appearance and histology sometimes confound our ability to make accurate diagnoses of the state of malignancy and grade. Conventional methods that combine history, plain radiographs, and histology may result in both under and over treatment. A method, which allows preoperative assessment of malignancy and grade would be valuable in developing a rationale plan for treatment. We examined the correlation between specific nuclear tracer avidity and malignancy in cartilage tumours.
*Methods:* Thallium-201 (Tl-201) and pentavalent dimercaptosuccinic acid (DMSAV) are two isotopes which when used as nuclear tracers, may provide enhanced information regarding the biologic activity of cartilage tumours. Our study examined 92 consecutive cartilage tumours (50 benign, 42 malignant), which were treated at our institution between 1996 and 2000 and analysed the correlation between Tl-201 and DMSAV scanning and malignancy and grade.
*Results:* We noted that patients with negative DMSAV scans (25) had benign tumours. 15 of 17 patients with positive TL-201 scans had malignant tumours. Patients with both positive DMSAV and TL-201 scans (11/13) had intermediate or high grade tumours. One of two patients with positive DMSAV and Tl-201 scans but with an apparently benign tumour demonstrated chromosomal changes consistent with chondrosarcoma. Four of thirteen patients with positive DMSAV and TL-201 scans developed metastases.
*Conclusion:* From our results we were able to develop an algorithm for the management of patients with cartilaginous tumours that aims to avoid over treatment of low grade tumours, or under treatment of high grade tumours. We conclude that functional nuclear scanning with DMSAV and Tl-201 is a valuable technique in the management of cartilaginous tumours.

### D42 3-D Imaging in Planning and Monitoring Biological Reconstructions in Orthopaedic Oncology: A Computer Assisted Approach. Future Perspectives and Preliminary Results

M. Manfrini, F. Taddei, D. Donati, C. Malaguti, S. Giacomini, M. Viceconti
*Istituti Ortopedici Rizzoli, Bologna, Italy*

Biological bone reconstruction in orthopaedic oncology always has to face two major problems: the pre-operative planning and the post-operative management, that are currently highly subjective due to a lack of specific instruments and heavily related to surgeon's experience. Aim of this work is to present a few research lines currently active at our institution to improve the management of reconstructive tumour surgery and to present some preliminary results. Up-to-date Medical Imaging and computer modelling technologies allow in principle the development of pre-operative planning tools with which the surgeon can navigate through a three-dimensional environment, exploiting the information derived from different imaging modalities. Starting from CT and MRI datasets, subject-specific finite element models (FEM) of bone segments can be created and used to predict the mechanical behaviour of the reconstructed bone. Subject-specific information on gait pattern and muscle activation strategies could be derived from the gait analysis and linked to the bone models to predict their behaviour during various physical activities evaluating the associated fracture risk. All these tools could provide the clinicians with quantitative information to help him in better planning surgery and rehabilitation programs. At present, the evaluation of the mechanical compliance of the reconstructed bone, and its evolution during the follow-up is almost impossible. A non-invasive technique is here presented with the preliminary results relative to two patients with biological femur reconstructions evaluated at different follow-up. Three-dimensional FEM of the reconstructed femurs and of the intact contralateral ones were created, starting from the CT dataset, and the peak loads during unprotected level walking were simulated. The simulation allows an accurate analysis of the stress pattern in the various femur regions and an evaluation of the risk of fracture in the reconstructed bone.

### D43 Value of Dynamic Contrast-Enhanced MR Imaging in Diagnosing and Classifying Peripheral Vascular Malformations

C.S.P. van Rijswijk, E. van der Linden, H.J. van der Woude, J.M. van Baalen, J.L. Bloem
*Leiden University Medical Center, Leiden, The Netherlands*

*Purpose:* To evaluate prospectively whether MR imaging, including dynamic contrast-enhanced MR, can be used to categorize peripheral vascular malformations and especially to identify venous malformations that do not need angiography.
*Methods:* Two observers correlated independently, in this blinded prospective study, MR imaging findings of 27 patients with peripheral vascular malformations with those of diagnostic angiography and additional venography. MR diagnosis of category, based on a combination of conventional and dynamic contrast-enhanced MR parameters, was compared with the angiographic diagnosis using gamma statistics. Sensitivity and specificity of conventional MR imaging and dynamic contrast-enhanced MR imaging in differentiating venous from non-venous malformations were determined.
*Results:* Excellent agreement between the two observers existed in determining MR categories (□-value of 0.99). Agreement between MR categories and angiographic categories was high for both observers (□-value of 0.97 and 0.92). Sensitivity of conventional MR imaging in differentiating between venous and non-venous malformations was 100%, while specificity was 24–33%. Specificity increased to 95% by adding dynamic contrast-enhanced MR imaging, but sensitivity decreased to 83%.
*Conclusion:* Conventional and dynamic contrast-enhanced MR parameters can be used in combination to categorize peripheral vascular malformations. Dynamic contrast-enhanced MR imaging allows diagnosis of venous malformations with high specificity. Treatment of vascular malformations is based on identification of these categories.

### D44 Hip and Sacroileac Joint Infiltration in Sarcoma Around the Pelvis Based on Imaging and Pathoanatomical Correlation Studies and its Implications on Surgical Strategy

N.J. Lindner[1], G. Gosheger[2], C. Gebert[1], T. Ozaki[1], W. Winkelmann[1]
*[1]University of Muenster, Muenster, Germany, [2]Klinik und Poliklinik Allgemeine Orthopaedie, Muenster, Germany*

The incidence and characteristics of tumour infiltration in sarcomas around the pelvis were analysed. 118 patients with a sarcoma (chondrosarcoma; 46, Ewing's sarcoma; 35, and osteosarcoma; 37) arising around the pelvis (Ileum; 51, acetabulum; 50, femur; 17) underwent surgical treatment. Preoperative computed tomography and magnetic resonance imaging was matched with histological findings in tumour specimens. Tumour infiltration into the hip joint was suspected based on imaging techniques in 29 patients due to articular disruption, diffuse signal change of the acetabulum or femoral neck, existence of tumour mass in the joint, and massive joint effusion. Of the 29 patients, 15 patients showed histologic tumour invasion. 12 of 31 chondrosarcomas, none of 12 Ewing's sarcomas, and 3 of 24 osteosarcomas infiltrated in the hip joint (p=0.008). Fifteen of 51 patients had a true sacral infiltration from pelvic sarcoma. Age (p=0.0570) and tumour grade (p=0.3014) were not correlated with joint infiltration. There was a significant difference of median volume of sarcomas between with and without infiltration (p=0.0127). 1/23 Ewing's sarcomas, 7/15 chondrosarcomas, and 7/13 osteosarcomas penetrated the sacroiliac joint into the sacrum (p=0.0016). Logistic regression test showed that diagnosis was the most important factor to influence sacral infiltration (p=0.0059). Of 10 tumours originating from the acetabulum, 9 penetrated through the osseous-ligamentous junction and one through the acetabular cartilage. In 5 proximal femur lesions, all infiltrated the joint through the femoral neck with or without through the ligamentum teres. Twelve tumours infiltrated through the posterior part of the joint, 2 tumours through the anterior part, and one large tumour through the unclear route. Resection in lesions around the pelvis at risk for sacroiliac or hip joint involvement need to be extended beyond the capsule through healthy bone to achieve a safe margin, especially in osteosarcoma and chondrosarcoma.

### POSTER PRESENTATIONS

### D45 Magnetic Resonance Guide (MRT) in Tumour Surgery- our Preliminary Experience

M. Salai, A. Garniek
*Chaim Sheba Medical Center, Tel Hashomer, Israel*

Appropriate surgical margins, are one of the mainstay of successful surgical oncology procedure. Preoperative planning and fluoroscopy plus the surgeon eyes are currently the navigation methods mostly employed. The introduction of MRI and recently the active MRI/MRT had opened a new era in intra-operative imaging, especially in tumour surgery. It offers ultimate accurate intraoperative imaging as a navigator to the surgeon. Though it has some drawbacks such as: narrow space and inconvenience to the surgeons, lack of bone penetrating instruments, slow sequence of images, etc. During the last 18 months we had used active MRI in the surgical treatment of 12 patients who suffered from tumours of the musculoskeletal system. Three patients had lipoma, 2 had recurrent liposarcoma, 3 patients had schwannoma, 2 patients had pigmented villo-nodular synovitis, one had neurofibroma and one ganglion cyst. In all these operation we could correlate the preoperative films with direct vision of the lesions in correlation to the MRI active navigation, and achieve appropriate margins (especially in the patients with liposarcoma). Gadolinium enhancement was also utilized intraoperatively in most cases. Our preliminary experience indicate that with further developments of the MR technology, non magnetic instruments, contrast materials, and surgical experience, active MRI assistance might become an integral part of orthopedic oncology surgery. This might lead to achievement of better surgical margins, lesser resection of non-tumourous tissues, less local recurrences, and presumably better survival of the patients.

### D46 Somatostatin Receptor Scintigraphy in Patients with Osteosarcoma

S. Ferrari[1], M. Dondi[2], S. Fanti[2], S. Zoboli[1], S. Giacomini[1], M. Mercuri[1], G. Bacci[1]
*[1]Istituti Ortopedici Rizzoli, Bologna, Italy, [2]Ospedale Maggiore, Bologna, Italy*

*Objective:* To investigate somatostatin-receptors in primary or metastatic lesions of patients with osteosarcoma.
*Methods:* 18 patients (median age 15, 6–48) with high-grade osteosarcoma (12 without metastases at diagnosis, 4 with synchronous metastases, and 2 with metachronous bone metastases). To detect somatostatin-receptors, somatostatin receptor scintigraphy (SRS) was used. SRS was carried out after i.v. injection of 200–250 MPq of 111 In-Pentreotide; images were recorded at 4 and 24 hours. All patients underwent 99mTc-MDP bone scan (BS). Scans were interpreted both with qualitative evaluation and with semi-quantitative image processing, by means of tumour-to-background ratio modification over time. SRS was considered as positive on the basis of tumour uptake degree and extent (in comparison with BS) and on the basis of increased tumour-to background ratio at 24 hours (in comparison to 4 hours).
*Results:* Overall, in the primary tumour SRS was positive in 13 (72%) patients: 10 (83%) of the 12 non metastatic patients, and 2 (50%) of those with synchronous metastases, and 1 (50%) with recurrent osteosarcoma. In the metastatic lesions, SRS was positive only in one (17%) case. No relation was found between SRS Results and sex, age, SAP level, and tumour volume. After primary chemotherapy, 15 patients underwent surgery of primary tumour: a good histologic response was found in 7 (64%) of the 11 SRS positive patients vs 1 (25%) of the 4 patients with negative SRS (p = 0.17).
*Conclusion:* In the vast majority of patients with osteosarcoma, primary tumour exhibits somatostatin receptors, whereas SRS is more frequently negative in the metastatic lesions. Further investigations are needed to verify whether a negative result of SRS is due to a low sensitivity of the technique or is related to biological differences in somatostatin receptors expression.

### D47 Imaging of Primary and Recurrent Sacral Tumours

E.R. Mousaev, A.B. Lukianchenco, T.K. Kharatishvili, M.D. Aliev
*Blokhin Cancer Center, Moscow, Russian Federation*

*Background:* Clinical symptoms of sacral tumours are characterized with absence of specificity and depend on nerve root or intrapelvic involvement and the aggressiveness of the lesion. Timely detection of diagnosis represents significant difficulties taking into consideration of rarity of sacral tumours.
*Methods:* From 1985 to 2001 57 patients with sacral tumours were treated in department of bone and soft tissue tumours. 38 patients underwent sacral resection. The local recurrences were observed at 7 patients. The most frequently histology type was chordoma – 22 patients (38,6%). Mean age was 35,4 years (18–74). Extraosseous extension was intrapelvic in 31 patients (54,4%), extrapelvic in 3 patients (5,3%) and both extra- and intrapelvic – in 23 (40,3%).
*Results:* On plain radiographs sacral tumours reflected the type of matrix produced although the findings can be subtle. The CT scan also revealed any matrix production. Any subtle periosteal reaction were best with CT scan, MRI was the best study for evaluation of the soft tissue and intramedullary and epidural extent of the lesion. Fluffy 'popcorn' calcifications were prominent in chondrosarcoma which extended into the intrapelvic or paraspinal soft tissues. Ewing's sarcoma and plasmacytoma were predominantly lytic. On plain radiograph chordoma caused destruction of sacral bone and enlargement of the neural foramina, areas of calcification were in 42% cases. Computerized tomography (CT) demonstrated bone destruction in 85% cases of chordoma, the calcific debris was noted in 80%. Invasion of the gluteal muscles was the best on MRI compared with CT scan. MRI imaged spinal canal invasion with dural displacement or occlusion. The direct sagittal images helped to determine tumour extent.
*Conclusion:* CT scan and MRI are more sensitive imaging techniques, than plain radiograph in the local recurrence detection of sacral tumours.

**D48 Dedifferentiated Chondrosarcoma: Image Guided Percutaneous Needle Biopsy.**
S. Mahroof, B. Mann, A. Saifuddin, J. Pringle, T.W. Briggs, S.R. Cannon
*Royal National Orthopaedic Hospital, London, United Kingdom*

*Objective:* Some low grade chondrosarcomas have the tendency to transform to a high grade tumour, typically an osteosarcoma, fibrosarcoma or malignant fibrous histiocytoma, a process termed dedifferentiation. The significance of this change lies in the management and prognosis of the tumour. Prognosis is often as for the more aggressive dedifferentiated area of the tumour, while management changes include the use of neo-adjuvant chemotherapy and radiotherapy. We describe five cases in which MRI was used to identify and biopsy areas of dedifferentiation in patients thought to have low grade chondrosarcoma.
*Methods:* In all the cases, MRI studies included a combination of sagittal and/or coronal T1 weighted spin echo and STIR sequences, together with an axial T2 weighted fast spin echo sequence with fat saturation. In low-grade and myxoid chondrosarcoma, MRI shows a classical lobulated, hyperintense appearances on T2 weighted sequences. However, in addition to this appearance, areas of lower signal intensity lacking in lobulation were also present. These areas were preferentially biopsied using CT guidance.
*Results:* Three of the five patients were male. One patient had diaphyseal aclasis. The age ranged between 34–71 years (mean age 59 years). The sites involved were the ilium, proximal femur, femoral diaphysis and two distal femoral lesions. In two patients the dedifferentiated component consisted of osteosarcoma and in the remaining three, it was malignant fibrous histiocytoma. All patients received chemotherapy as part of their treatment and four of the five also had definitive surgery.
*Conclusion:* The cases illustrate the importance of careful imaging assessment of cartilage tumours and the value of image guided needle biopsy by a radiologist with particular experience of bone tumour imaging.

**D49 Prognostic Factors in Localized High-Grade Osteosarcoma. The Role of Imaging Techniques**
G.N. Matchak[1], A.B. Lukianchenko[1], N.V. Kochergina[1], O.G. Zimina[1], O.V. Senko[2], A.V. Kuznetsova[2], M.D. Aliev[1]
*[1]Blokhin Cancer Center, Moscow, Russian Federation, [2]Computer Center of RAS, Moscow, Russian Federation*

*Objective:* Pre-treatment stratification of patients with osteosarcoma remains difficult. The objective of this study was to determine the prognostic significance of patient and disease-related prognostic factors at diagnosis.
*Methods:* Between 1983 and 1998, 137 patients received i.a. DOX, 36 Gy, surgery and chemotherapy (protocol A), 169 received 3–4 cycles of i.a. DOX or DDP, surgery and chemotherapy (protocol B). Sex, age, tumour site, anamnesis, Enneking stage, absolute (AV) and relative (RV) volume, growth rate, radiographic appearance, morphology, vascularity, and alkaline phosphatase (AP), were analysed as prognostic variables. Anteroposterior radiograph, CT, MR, and angiography were used to obtain an integrate information about tumour. Cox regression and pattern recognition technique were used for multivariate analysis.
*Results:* Protocol A: survival was related to Enneking staging (p=0.001), AV (p=0.002), growth rate (p=0.006), and AP (p=0.027). The presence of large areas of low attenuation in soft-tissue component seen on CT scans (haemorrhage/necrosis), high signal with all MR pulse sequence (haemorrhage), low signal intensity on T1-weighed images and high signal intensity on T2-weighed MR images (necrosis) correlated with poor prognosis (p=0.05). Enneking stage, AV, RV, growth rate, and the ratio of lytic and blastic components within bone were significant in multivariate analysis. Protocol B: RV (p=0.03), AV (p=0.0001), growth rate (p=0.0014), and AP, (p=0.0003) were significant in univariate analysis. Tumours with homogenous non- or low mineralised soft-tissue masses had more favourable prognosis in comparison with tumours containing opacities (p=0.00008). Isolated lamellated periosteal reaction was associated with more favourable outcome versus the other reactions (p=0.01). Presence of large nonvascular areas was the indicator of poor prognosis, (p=0.03). AV, growth rate and degree of soft-tissue masses ossification were significant in multivariate analysis.
*Conclusion:* Prognostic factors varied in different protocols. Modern imaging techniques gave additional prognostic information useful for pre-treatment identification of high-risk patients.

**D50 Soft Tissue Sarcoma Involving Bone or Neurovascular Structures**
B. Y. Bokhyan, R. M. Karapetian
*Russian Cancer Research Center, Moscow, Russian Federation*

*Purpose:* To determine if x-ray computered tomography (CT) and magnetic resonance tomography (MR) imaging findings of soft tissue sarcoma involving bone or neurovascular structures allow prediction of local recurrence, distant metastasis or survival. CT or MR images of 150 patients with soft tissue sarcomas were retrospectively reviewed for tumour involving bone or major vessels or nerves. The imaging findings were correlated with local recurrence, distant metastasis and survival after treatment.
*Methods & Results:* The most common location of the primary (110–74%) and recurrent tumours was the lower extremity (75%). On CT or MR images, bone invasion occurred in 30 patients – 20%, vessel encasement 45 patients – 30% and majors nerve encasement in 10 patients – 6.6%. In patients with and those without bone invasion, frequencies of disease related death were significantly different; frequencies of local recurrence or distant metastasis were not significantly different. In patients with and those without major vessel or major nerve encasement, there were no significant differences between frequencies of local recurrence, distant metastasis or survival.
*Conclusion:* Bone invasion on CT or MR images was predictive of decreased survival. CT or MR imaging findings of or neurovascular involvement otherwise appear to be more important for planing surgery than for predicting local recurrence, distant metastasis or survival.

**D51 Color Doppler Ultrasound as a Prognostic Factor in Osteosarcoma**
J.A.M. Bramer[1], F.M. Gubler[1], M. Maas[1], J. Bras[1], J. De Kraker[2], J.W. Vd Eijken[1], G.R. Schaap[1]
*[1]Academic Medical Centre, Ouderkerk A.D. Amstel, The Netherlands, [2]Emma Kinderziekenhuis, Amsterdam, The Netherlands*

*Introduction:* The most important prognostic factor in osteosarcoma, cell necrosis after chemotherapy, can only be assessed after resection. It would be advantageous to be informed about prognosis at an earlier stage. Color Doppler Ultrasound (CD-US) can asses tumour vascularity. Goal of the study was to establish whether CD-US, performed before and after chemotherapy, can predict cell necrosis and survival.
*Methods:* In a prospective study 21 consecutive patients with osteosarcoma were included. CD-US was performed before and after chemotherapy. Flowspectrum of the feeding artery was assessed. Resistive Index (RI) calculated and compared with the corresponding contralateral artery. The highest flow rate in the tumour was assessed (Peak Systolic Velocity, PSV). Good response was defined as >19% decrease of PSV and normalizing of RI. After resection CD-US results were compared with celnecrosis (EOI standard) and patient survival.
*Results:* There was a good CD-US response in 9 patients, 6 of which had a good histologic response. A bad CD-US response was seen in 10 patients, 1 of them was not operated because of progressive disease during chemotherapy. The other 9 all showed a bad histology response, as did 2 with contradictive US-parameters. With a median follow up of 36 months (12–67) 6 of the 9 US-responders were without evidence of disease, so were the 2 with contradictive parameters. In the non-responder group 6 had died of disease, 1 suffered recurrence and 3 were without evidence of disease.
*Conclusion:* No significant correlation of CD-US with survival could be assessed in this study. With Color-Doppler-Ultrasound cell necrosis after chemotherapy in osteosarcoma can be predicted before resection. CD-US could be used in monitoring and possibly adjusting chemotherapy and in planning and timing of operation.

**D52 Thallium – 201 Scans is a Predictor of Response to Preoperative Radiotherapy in Soft Tissue Sarcoma**

P. Choong[1], T. Kunisada[1], G. Powell[1], S. Schlict[1], R. Hicks[2], J. Slavin[1]
[1]*St. Vincent's Hospital, Melbourne, Australia*
[2]*Peter MacCallum Cancer Institute, Melbourne, Australia*

*Background:* Thallium-201 is a potassium analogue and its uptake into cells is enhanced in hypermetabolic tissue because of the sodium potassium ATPase pump. Thallium scintigraphy in patients with malignant soft tissue tumours were evaluated to determine whether the images correlated with histologic response to preoperative radiotherapy.
*Methods:* We studied 54 patients with non-metastatic, malignant soft tissue tumours diagnosed between 1996 and 2001. Of these, 16 patients had previous incomplete excisions. Qualitative analysis of early (phase 1) and late (phase 2) scintigraphic images before and after preoperative radiotherapy were compared with the degree of tumour necrosis determined histologically.
*Results:* In those patients with primary soft tissue tumours, all 22 patients with biopsy proven soft tissue tumours who showed no-change in thallium scintigraphy had tumour necrosis of 80% or less. 13 of 16 patients who showed an improvement in thallium scintigraphy had tumour necrosis of 95% or more. In the incompletely excised group, 3 patients (with grade 1 retention in phases 2) who showed no-change in thallium scintigraphy had tumour necrosis of 45% or less; 8 patients who had improvements in their thallium scintigraphy studies had no residual tumour; 5 patients (with grade 0 retention in phase 2 pre and post radiotherapy) who had no-change in thallium scintigraphic imaging had no residual tumour.
*Conclusion:* Thallium scintigraphy is a readily available investigative tool, which should be used in conjunction with other imaging modalities in the assessment of primary and incompletely excised malignant soft tissue tumours, as it appears to predict histological tumour response to preoperative radiotherapy.

**D53 New Methods for Diagnosis of Giant Cell Tumour**

B.L. Lehner[1], D-S.A. Dimitrakopoulou-Strauss[2], L.M. Libicher[3], B.L. Bernd[1]
[1]*Orthopedic university clinic, Heidelberg, Germany,* [2]*DKFZ, Heidelberg, Germany,* [3]*Heidelberg university, Heidelberg, Germany*

*Purpose:* In diagnosis of primary and especially recurrent giant cell tumours conventional methods show limited accuracy. Therefore the value of dynamic magnetic resonance imaging (MRI) and of positron emission tomography (PET) should be examined in our study.
*Methods:* In 9 patients with bone tumours dynamic contrast enhanced MRI and in 9 patients dynamic PET studies using F18-Fluorodeoxyglucose (FDG) for assessment of FDG metabolism was performed. In 4 patients there was a local recurrent tumour, in 1 patient pulmonary metastases of the tumour. In all cases giant cell tumour was proved by histology.
*Results:* All giant cell tumours showed a unique perfusion pattern with a steep slope and maximum intensity value followed by an early and rapid washout phase in MRI. The same pattern appeared in local recurrent tumours. In FDG-PET the metabolism of FDG in the tumours was highly increased with a mean standardized uptake value (SUV) of 4.7. Muscle tissue showed a mean SUV of 0.5, bone tissue of the contralateral side 0.3. Increased SUV values >4 also could be found in local recurrent tumours and in pulmonary metastases.
*Conclusion:* Giant cell tumours showed a characteristic perfusion pattern in dynamic MRI and FDG-PET. These methods appear to be helpful in diagnosis of primary tumours as well as local recurrences.

